# Delayed extensive lumbar sub-dural effusion following discectomy - Clinical imaging and case report

**DOI:** 10.1051/bmdcn/2017070106

**Published:** 2017-03-03

**Authors:** Arun-Kumar Kaliya-Perumal, Meng-Ling Lu, Fu-Cheng Kao, Chi-Chien Niu

**Affiliations:** 1 Department of Orthopaedic Surgery, Spine Division, Bone and Joint Research Centre, Chang Gung Memorial Hospital Taoyuan 333 Taiwan; 2 College of Medicine, Chang Gung University Taoyuan 333 Taiwan; 3 Department of Orthopaedic Surgery, Melmaruvathur Adhiparasakthi Institute of Medical Sciences and Research Melmaruvathur Tamil Nadu 603319 India; 4 Department of Orthopaedic Surgery, Spine Division, Chang Gung Memorial Hospital Kaohsiung 833 Taiwan

**Keywords:** Subdural effusion, Pseudomeningocele, Dural Tear, Incidental Durotomy, Cerebrospinal fluid

## Abstract

Incidental durotomy during lumbar spine surgery is a commonly reported complication. Those presenting with cerebrospinal fluid (CSF) leak are usually recognized and repaired intraoperatively. In some circumstances, it may either be unrecognised or occur as a delayed complication. Such delayed occurrences cannot be predicted and its management remain a challenge to the surgeon, especially when it presents as a subdural effusion. We report a 55-year-old man who underwent mini open lumbar discectomy through left side for a prolapsed L4-L5 disc. Recurrent worsening radicular symptoms along with a palpable cystic swelling at the previous surgical site became eminent, three months after surgery. MRI revealed distinctive anterior translation of all rootlets with subdural fluid collection posterior to it, within a normally placed dura, extending from L1 to L5 levels. A concomitant pseudomeningocele with a fistulous tract was also evident. Draining of pseudomeningocele with widening of previous laminotomies revealed a dural defect of less than 0.5 cms that prompted the CSF leak. Subdural effusion was drained following which the defect was repaired with inlay polyester urethane dural substitute patch and augmented with fibrin sealant. Symptoms regressed and follow up was uneventful. Occurrence of sub-dural effusion in lumbar spine is inevitably uncommon. We advise to suspect this condition in patients with recurrent symptoms following satisfactory lumbar decompression surgeries. Recognising this condition, followed by appropriate drainage of subdural effusion and direct repair of the dural defect is highly recommended for a better prognosis.

## 1. Introduction

Subdural effusion occurring in the lumbar region was earlier described in 1973 [1]. It can be described as subdural loculation or collection of cerebrospinal fluid (CSF) in the dura - arachanoid interface. Incidental durotomies during lumbar spine surgery is a commonly reported complication and a primary repair by direct suturing is widely recommended [2–4]. Rarely, patients may develop delayed CSF leak even if there was no dural tear noticed during initial surgery. Such CSF leak can be attributed to an unrecognized dural tear or to a de novo delayed dural tear [5]. This report depicts a rare occurrence of subdural effusion coupled with a large pseudomeningocele, late after initial satisfactory lumbar decompression surgery. We portray its unique imaging characteristics and a novel approach to management.

## 2. Case Report

**Fig. 1.**
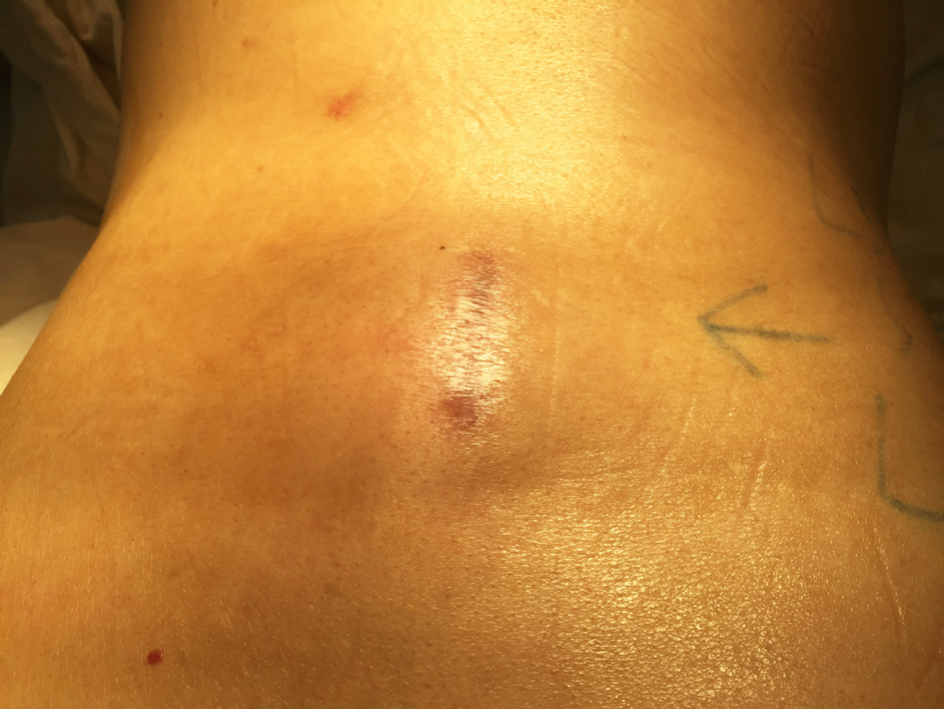
Pre-operative clinical picture showing the swelling noticed over previous surgical site.

A 55-year-old male was initially encountered with clinical and radiological indications for lumbar decompression surgery. A mini open discectomy through left L4/L5 laminotomy was performed. Intraoperative and initial post-operative period was uneventful and patient improved symptomatically. He developed recurrent radicular symptoms, 3 months following initial surgery. Symptoms included frequent headache and dizziness associated with difficulty in walking. Clinical examination revealed a positive straight leg raising test on left side with no neurological deficit. A 5 x 5 cm palpable cystic swelling was present in the previous surgical site (Fig. 1).

**Fig. 2:**
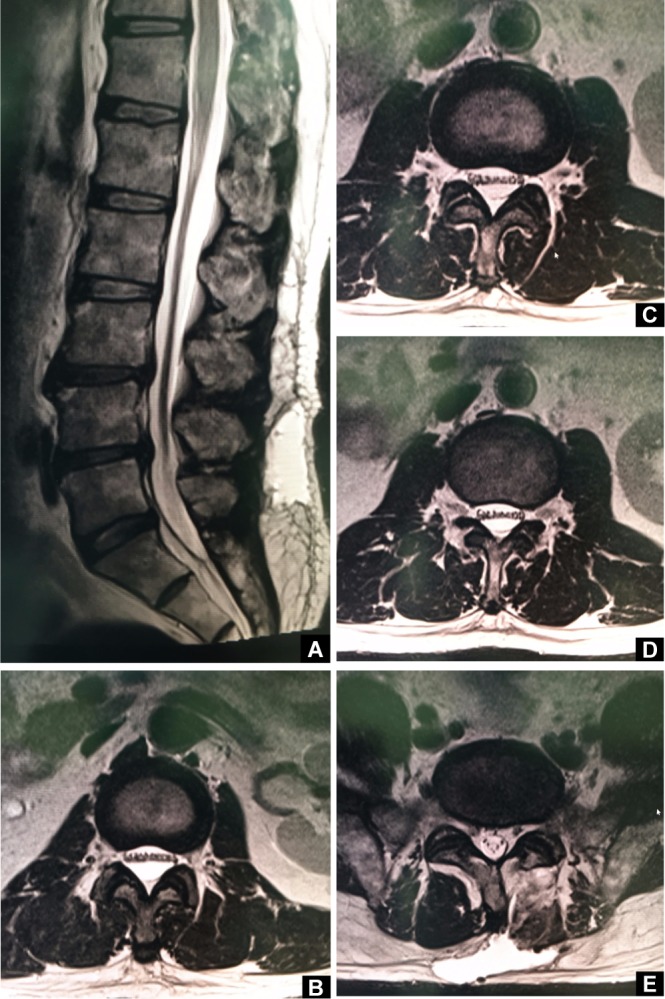
MRI images. (A) MRI sagittal cut depicting anterior translation of cauda equina from L1-L5 levels. (B) MRI axial cut at L1-L2 disc level showing unusual anterior linear arrangement of rootlets within a normally placed dura. (C) MRI axial cut at L2-L3 disc level. (D) MRI axial cut at L3- L4 disc level. (E) MRI axial cut at L4-L5 disc level showing pseudomeningocele with a fistulous tract.

MRI was repeated and sagittal cuts revealed abnormal distinctive anterior translation of cauda equina from L1 to L5 levels (Fig. 2a). Axial cut images showed anterior translation of all rootlets that were arranged in a linear pattern within a normally placed dura (Fig. 2b, 2c, 2d). Subdural accumulation of fluid posterior to the rootlets was certain as the dura was clearly visible adjacent to posterior wall of the spinal canal unlike epidural accumulations where the dural is pushed forward. This obvious subdural collection was monotonous from L1 to L4 levels and less obvious in L5 level. We hypothesize it as CSF accumulation since it was similar in intensity compared to CSF in both T1 and T2 weighted images. A large pseudomeningocele communicating to the previous laminotomy site through a fistulous tract was also evident (Fig. 2e). This MRI picture was pathognomonic to confirm that the collection of fluid was subdural and not extradural. A diagnosis of extensive lumbar subdural effusion associated with a pseudomeningocele was made.

**Fig. 3:**
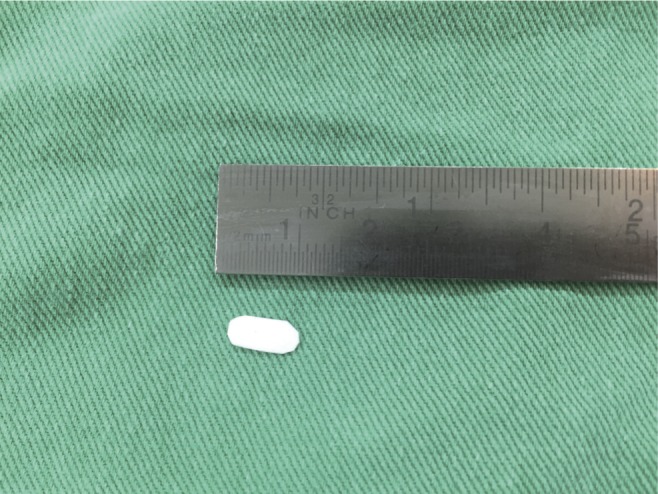
Trimmed Polyester urethane dural substitute patch which was inlaid within the dural defect.

After draining the pseudomeningocele, a fistulous tract was found that connected it to the laminotomy site. Widening of previous left sided L4 and L5 laminotomies were done and a dural defect of less than 0.5 cms was identified just above the shoulder of L5 nerve root that prompted the leak. Subdural effusion was drained by slightly widening the dural defect. The drained fluid from the subdural space was xanthochromic in appearance. After satisfactory drainage of subdural effusion, the arachanoid membrane and dura were brought together and a polyester urethane patch was used as an inlay dural substitute which was secured to the ends of the dural defect using 7-0 prolene (Fig. 3). A fibrin sealant was used to augment the dural repair. No further leakage of CSF was noted intraoperatively.

Symptomatic improvement in our patient was noted with no complaints of headache or dizziness in the days following surgery. Straight leg raising test was negative on both sides on the first postoperative day. Neurological status remained normal. Rehabilitation protocols were well tolerated and the patient was discharged on the 5^th^ Postoperative day. A review was scheduled every week in the first postoperative month and every month thereafter until final follow up. Patient was back to full functional status by 3 months and remained asymptomatic for rest of the follow up with no clinical or radiological recurrence.

## 3. Discussion

Incidental durotomies may remain undiagnosed when there is no obvious CSF leak, especially if the arachanoid membrane is intact. It may later open due to an increased intradural pressure during recovery [6]. Rarely, a de novo delayed dural tear can occur late after surgery due to a bony spicule projecting into the spinal canal that erodes and punctures the dura [5, 7]. During the initial surgery in our patient, we did not notice any CSF leakage. So, the later presentation may be due to a missed tear or a de novo delayed dural tear, but neither of it can be predicted nor substantiated [5]. Chronic CSF leak causes symptoms of headache and dizziness [8, 9]. Presence of associated radicular symptoms imply the possibility of canal stenosis. If stenosis is not due to a recurrent disc, other lesions inside the spinal canal such as subdural hematoma, subdural effusion, subdural hygroma or arachanoid cyst should be considered [10–14].

Repeating the MRI study is necessary for diagnosis in these cases [10–12]. MRI presentation as seen in our patient showing extensive anterior translation with monotonous linear arrangement of rootlets was never illustrated in the past. The xanthochromic appearance of fluid from the subdural space may be due to a previous hematoma which ultimately became lysed [15]. Some authors describe that there is no virtual space between the dura and the arachanoid membrane but their interface is filled with neurothelial cells [16]. These neurothelial cells can break due to mechanical forces, thus giving way for a subdural space to be formed where CSF can enter [17]. CSF entering here can dissect open the space and become extensive as in our patient.

Recognising this condition in an MRI is very important because just draining the pseudomeningocele and closing the dural defect will not relieve the pressure symptoms caused due to the subdural effusion. The effusion needs to be adequately drained, also the dura along with arachanoid membrane needs to be repaired to prevent re-seepage of CSF into this space. Care should be taken not to tensely bring the dural ends together as it may further tear the dura. It is for this purpose we used a polyester urethane patch as a dural substitute so that the need for bringing the dural ends together is overcome [18]. We additionally augmented our repair with a fibrin sealant [19, 20].

## 4. Conclusion

This report depicts a rare complication following satisfactory lumbar decompression surgery. Delayed CSF leak causing subdural effusion or other complications cannot be predicted if the initial surgery was uneventful. Intraoperative meticulous handling of dura may prevent such complications. Repeating MRI study is the only key to diagnosis. Direct repair of the dural defect is highly recommended for a better prognosis.

## Conflicts of interest

The authors have no conflicts of interest relevant to this article.
